# Transcriptome Profiling of Radish (*Raphanus sativus* L.) Root and Identification of Genes Involved in Response to Lead (Pb) Stress with Next Generation Sequencing

**DOI:** 10.1371/journal.pone.0066539

**Published:** 2013-06-20

**Authors:** Yan Wang, Liang Xu, Yinglong Chen, Hong Shen, Yiqin Gong, Cecilia Limera, Liwang Liu

**Affiliations:** 1 National Key Laboratory of Crop Genetics and Germplasm Enhancement, Engineering Research Center of Horticultural Crop Germplasm Enhancement and Utilization, Ministry of Education of P. R. China; 2 College of Horticulture, Nanjing Agricultural University, Nanjing, P. R. China; 3 School of Earth and Environment, and The UWA’s Institute of Agriculture, The University of Western Australia, Perth, WA, Australia; National Taiwan University, Taiwan

## Abstract

Lead (Pb), one of the most toxic heavy metals, can be absorbed and accumulated by plant roots and then enter the food chain resulting in potential health risks for human beings. The radish (*Raphanus sativus* L.) is an important root vegetable crop with fleshy taproots as the edible parts. Little is known about the mechanism by which radishes respond to Pb stress at the molecular level. In this study, Next Generation Sequencing (NGS)–based RNA-seq technology was employed to characterize the *de novo* transcriptome of radish roots and identify differentially expressed genes (DEGs) during Pb stress. A total of 68,940 assembled unique transcripts including 33,337 unigenes were obtained from radish root cDNA samples. Based on the assembled *de novo* transcriptome, 4,614 DEGs were detected between the two libraries of untreated (CK) and Pb-treated (Pb1000) roots. Gene Ontology (GO) and pathway enrichment analysis revealed that upregulated DEGs under Pb stress are predominately involved in defense responses in cell walls and glutathione metabolism-related processes, while downregulated DEGs were mainly involved in carbohydrate metabolism-related pathways. The expression patterns of 22 selected genes were validated by quantitative real-time PCR, and the results were highly accordant with the Solexa analysis. Furthermore, many candidate genes, which were involved in defense and detoxification mechanisms including signaling protein kinases, transcription factors, metal transporters and chelate compound biosynthesis related enzymes, were successfully identified in response to heavy metal Pb. Identification of potential DEGs involved in responses to Pb stress significantly reflected alterations in major biological processes and metabolic pathways. The molecular basis of the response to Pb stress in radishes was comprehensively characterized. Useful information and new insights were provided for investigating the molecular regulation mechanism of heavy metal Pb accumulation and tolerance in root vegetable crops.

## Introduction

The problem of heavy metal pollution is becoming a major worldwide ecological concern due to its potential adverse impacts on human health through the food chain [1], [2]. Lead (Pb) is one of the most toxic heavy metals and can be easily taken up by plants and accumulated in different parts [3], [4]. The sources of Pb contamination not only include roots in natural processes but also derive anthropogenic activities, such as exhaust fumes of automobiles, emissions from industrial factories and land applications of agrochemicals including insecticides, herbicides and fertilizers [3], [5].

Lead is absorbed by plants mainly through the roots, and in most plant species the Pb content in organs tends to decrease in the order of root, leaves, stem, inflorescence and seeds [6]–[9]. Many previous studies reported that Pb overload in plants causes a range of negative effects, and the visually nonspecific symptoms of Pb toxicity are rapid inhibition of seed germination, stunted growth of the plant and chlorosis [1], [4]. Additionally, Pb can cause oxidative damage by stimulating the formation of free radicals and reactive oxygen species, resulting in oxidative stress and DNA damage [10]–[12].

Radish (*Raphanus sativus* L.), belonging to the family *Brassicaceae*, is an economically important vegetable crop with an edible taproot [13]. It was reported that radish roots could accumulate large amounts of lead (Pb), which could cause a potential health risk in polluted conditions [6], [14]. Considerable efforts have been invested into investigating Pb stress in radishes, especially its accumulation, translocation, physiological and metabolic variations, and cell deposition [6], [15]–[16]. However, little is known about the mechanism of radish plant responses to Pb stress at the molecular level,i.e.gene expression variations under Pb stress.

The next-generation sequencing (NGS) technologies including Roche/454’s sequencing [17],Illumina/Solexa’s sequencing technology (San Diego, California, USA), and Applied Biosystems’ SOLiD sequencing technology have provided novel opportunities for accelerating genome projects such as whole-genome re-sequencing for variation analysis, RNA sequencing (RNA-seq) for transcriptome and non-coding RNAome analysis [18], [19]. Among them, NGS-based RNA-seq for transcriptome methods can permit simultaneous acquisition of sequences and can be used to characterize genes, detect gene expression, identify and quantify rare transcripts without prior knowledge of a particular gene, and provide information regarding alternative splicing and sequence variations in identified genes [20]. In recent years, RNA-seq has been proven as a powerful method for understanding the complexity of gene expression and regulation networks in several plant species responding to many kinds of biotic and abiotic stresses, such as cotton [21], chinese cabbage [22], chickpea [23], maize [24], potato [25], *Ammopiptanthus mongolicus* [26] and soybean [27].

Previous studies indicated that plant in different stress responses is controlled by a range of gene regulatory mechanisms which could act together in various response and defense systems. Transcription factors, transport proteins and some other critical genes involved in certain signal transduction and secondary metabolite pathways were considered to be common stress-related transcripts activated in both biotic and abiotic stresses[27]–[29]. However, there were some unique genes involved in response to a specific stress [28]. For example, plants counteract the heavy metal stress by adopting both common and some specific means to defense and detoxifying the excessive concentration occurring in their surroundings, which includes generating metal ions signal sensing and transduction related pathways, activating numerous metal transporters and modulating related transcription factors[30]–[33]. It was found that the major activated genes not only involved in common stress-induced responses but also in some specific pathways including sulfur assimilation, glutathione (GSH) metabolism, IAA and jasmonic acid (JA) biosynthesis in *A. thaliana* under Pb treatments [34].

Although the molecular regulation mechanisms and massive candidate genes involved in various stress conditions have been successfully revealed by employing NGS technologies based on two main platforms including Roche/454 and Solexa/Illumina, their applications in investigating the dynamically transcriptional changes in response to heavy metal Pb stress in radishes has not been reported. The present study is aimed to elucidate the molecular regulation mechanisms and the critical genes involved in regulating radish responses to Pb stress. NGS-based Illumina paired-end Solexa sequencing platform was employed to characterize the fleshy taproot *de novo* transcriptome of two radish root cDNA samples, one untreated control (CK) and one Pb-stressed with Pb(NO_3_)_2_ at 1000 mg L^−1^(Pb1000). Furthermore, an overall gene expression analysis was compared between CK and Pb1000 based on the assembled *de novo* transcriptomes. From that, a large set of radish transcript sequences that could be used to discover root-specific functions were generated, an abundance of differentially expressed Pb-responsive genes were quantified, and the enriched networks for regulating heavy metal Pb stress were acquired in radishes. Additionally, expression profiling of some differentially regulated genes were validated by qRT-PCR. The molecular basis of the response to Pb stress was first comprehensively characterized in radish, and the resulting information would facilitate further investigation of the mechanisms of Pb accumulation/tolerance in plants and the effective management of Pb contamination in vegetable crops by genetic manipulation.

## Results

### Illumina Sequencing and *de novo* Transcriptome Assembly of Radish Roots

**Figure 1:**
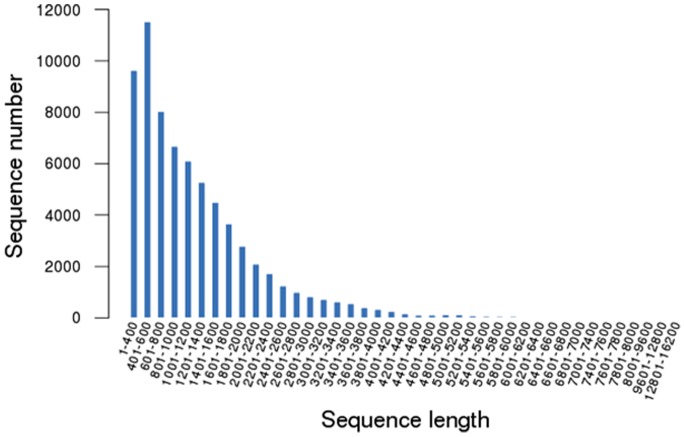
The length distribution of the assembled transcripts.

A large volume data was generated with the Illumina HiSeq 2000 sequencing the two radish cDNA libraries, CK, an untreated control and Pb1000, a Pb-stressed with Pb(NO_3_)_2_ at 1000 mg L^−1^. According to Illumina’s RNA transcriptome sequencing, a sequence can generate 2×101 basepairs (bps) from each Paired-End (PE) of a cDNA fragment. After removing the low quality reads and trimming off the adapter sequences, 26,381,880 (CK) and 22,535,988 (Pb1000) high-quality, clean paired-end sequencing reads with a total of 2,636,208,012 and 2,251,686,397 nucleotides were obtained, respectively, for the two pools. Because no appropriate reference genome sequence was available for radishes, a *de novo* assembly program Trinity [35] was selected for *de novo* assembly of all the 48,918,868 clean reads. Then, we combined all the assembling transcripts with sequencing of 31, 4823 radish expressed sequence tags (ESTs) and 17,187 unigenes from the NCBI database (http://www.ncbi.nlm.nih.gov/). Finally, a total of 68,940 assembled transcripts including 33,337 unigenes were obtained with the contig size ranging from 306 to 16,101 bp in transcript length and the average size of 1,226.63 bp (Table 1). The size distribution of the assembled transcripts is shown in Figure 1.

**Table 1:** Overview of the sequencing and assembly.

Items	Number
Total genes	33,337
Max contig length(bp)	16.101
Min contig length(bp)	306
Whole dataset lengths(bp)	84,563,748
Average contig lengths(bp)	1,226.63
Total isogenes	68,940
N50	1,262
N90	437

### Function Annotation and Classification of the Assembled Transcripts

**Figure 2:**
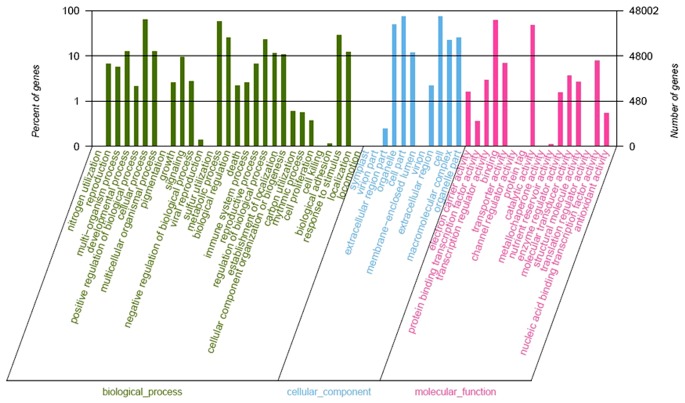
Gene ontology classification of assembled transcripts.

Gene Ontology (GO) assignments were used to classify the functions of the predicted radish root genes under the heavy metal lead (Pb) stress. Based on sequence homology, 46,966 transcript sequences (68.12% of all assembled sequences) were assigned at least one GO term, including 57 functional groups at the second level (Figure 2). Among which, ‘cell’ (34,290 sequences, 73.01%), ‘cell part’ (36,289 sequences, 73.01%) and ‘organelle’ (23,439 sequences, 49.91%) terms were dominant in the cellular component, while ‘cellular process’ (30533 sequences, 65.01%),‘metabolic process’ (27,531 sequences, 58.62%) and ‘response to stimulus’ (14,392 sequences, 30.64%) were the most highly represented under biological process, and ‘binding’ (28856 sequences, 61.44%) and ‘catalytic activity’ (21742 sequences, 46.29%) were the most abundant subcategories for the molecular function. Furthermore, only a few transcripts were clustered in terms of ‘virion part’, ‘protein tag’, ‘translation regulator activity’, ‘metallochaperone activity’, ‘channel regulator activity’ and ‘sulfur utilization’.

**Figure 3:**
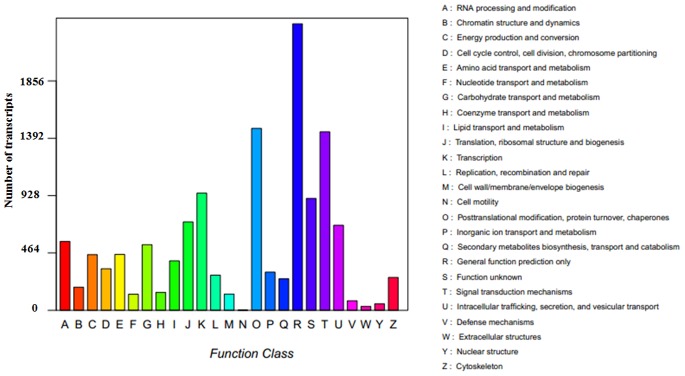
COG function classification of assembled transcripts.

To further evaluate the completeness of the *de novo* assembly transcriptome and predict possible functions, all assembled transcripts were compared against the Cluster of Orthologous Groups (COG database) for the analysis of phylogenetically widespread domain families. The results revealed 22,518 sequences with significant homology and assigned them into the appropriate COG clusters. These COG classifications were grouped into 23 functional categories (Figure 3). The five largest categories were ‘general function prediction only’ (30.05%), ‘signal transduction mechanisms’ (15.89%), ‘transcription’ (14.70%), ‘post translational modification, protein turnover, chaperones’ (13.59%), and ‘function unknown (12.69%).

The Kyoto Encyclopedia of Genes and Genomes (KEGG) is a database resource for the systematic understanding of high-level gene functions in terms of networks of the biological system, such as the cell, organism and ecosystem, from molecular-level information (http://www.genome.jp/kegg/). The assembled transcript sequences were searched against the KEGG database using BLASTX with a cut-off E-value of 10^−5^ to identify the biological pathways activated in the roots of radishes in response to heavy metal Pb stress. Of a total of 48,864 annotated sequences, 19,480 had a significant match in the database and were assigned to 296 KEGG pathways (Table S1). The major pathways were ‘metabolic pathways [ko01100]’, ‘biosynthesis of secondary metabolites [ko01110]’, ‘microbial metabolism in diverse environments [ko01120]’, ‘ribosome [ko03010]’ and ‘plant hormone signal transduction [ko04075]; the sequence numbers and percentage of sequences assigned to each pathway were 4,269 (28.20%), 1,954 (12.91%), 1,059 (7.21%), 839 (5.54%) and 629 (4.15%), respectively.

### Differentially Expressed Genes Involved in the Response to Pb Stress in Radish Roots

**Figure 4:**
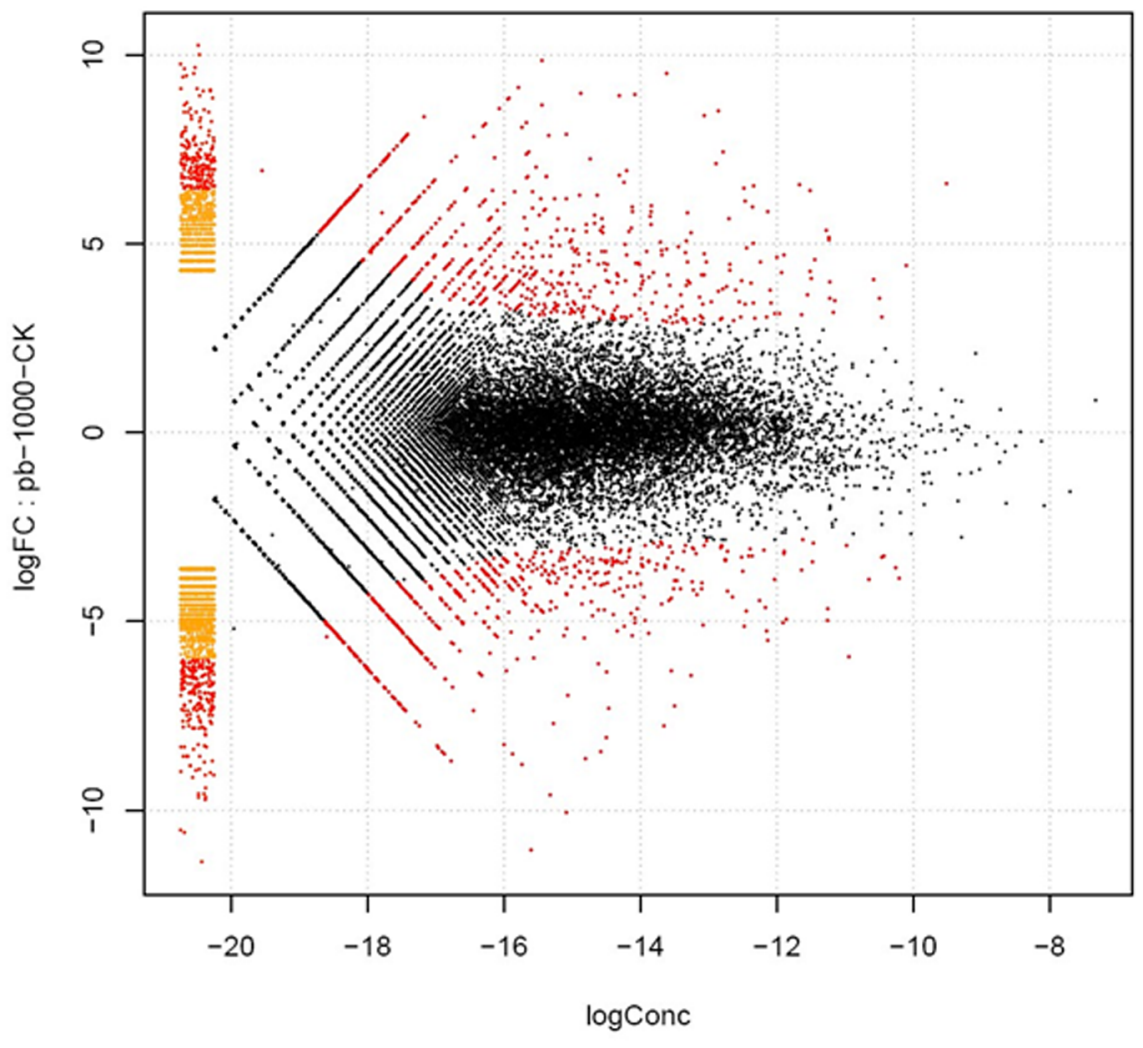
Volcano plot of gene expression difference between Pb1000 and CK samples.

To investigate the changes in gene expression and understand the critical genes in radish roots responding to the stress of heavy metal Pb, clean reads of the Pb1000 and the CK libraries were respectively mapped to the *de novo* assembly transcriptome reference sequences with Bowtie [36]–[37] and were assigned to unigenes and isoforms with the RSEM (RNA-Seq by Expectation Maximization) software [38]. The assigned unigene and isoform expression levels were calculated using a normalizing statistic called FPKM (fragments mapped per kilobase of exon per million reads mapped), which provides a measure of expression level that accounts for variation in gene length [39]. The differentially expressed genes (DEGs) (including unigene and isoform) were determined with a log-fold expression change (log FC) greater than 2 or less than –2 using a greater statistically significant value (*P*<0.005) as well as false discovery rates (FDR <0.001). From that, a total of 4,614 DEGs (1,398 unigenes) were detected between the two libraries, and these DEGs included both upregulated (2,154 transcripts) and downregulated genes (2,460 transcripts) under the Pb treatment (Figure 4).

### Gene Ontology (GO) and Pathway Functional Enrichment Analysis of the DEGs

All of the DEGs were performed on GO function and pathway enrichment analysis with a hypergeometric test and Bonferroni Correction [40]. GO terms significantly enriched in the DEGs were identified using goatools (https://github.com/tanghaibao/goatools). According to GO functional enrichment analysis, a total of 270 terms for the upregulated transcripts were significantly enriched at a Bonferroni-corrected *P*-value ≤0.05, while for downregulated ones only 107 terms were enriched. The GO terms predominantly enriched for the upregulated transcripts were related to biological processes including ‘defense response by callose deposition in cell wall’ (GO:0052544), ‘defense response by cell wall thickening’ (GO:0052482), ‘response to oxygen levels’ (GO:0070482), ‘glycoside catabolic process’ (GO:0016139), and ‘glutathione conjugation reaction’ (GO:0006803), and those related to molecular function include ‘glutathione transferase activity’(GO:0004364) (Table S2). The predominant GO terms identified as enriched among the downregulated transcripts related to ‘catalytic activity’ (GO: 0003824) in molecular functions, such as ‘cellulose synthase (GDP-forming) activity’ (GO:0016761), ‘carbon-oxygen lyase activity, acting on polysaccharides’ (GO: 0016837), and ‘cell wall organization or biogenesis’ (GO:0071554) in biological processes such as ‘cellular cell wall organization (GO:0007047) and cell wall modification (GO:0042545) (Table S3). The KEGG Orthology-Based Annotation System (KOBAS) was employed to find most statistically significantly enriched pathways for all the DEGs, which revealed that 13 and six pathways were observed significantly enriched for up- and down-regulated DEGs, respectively. As shown in Table 2, the pathways predominantly enriched for the upregulated transcripts were related to xenobiotics biodegradation and metabolism, such as drug metabolism-cytochrome P450 [ko00982], metabolism of xenobiotics by cytochrome P450 [ko00980], polycyclic aromatic hydrocarbon degradation [ko00624], aminobenzoate degradation [ko00627] and bisphenol degradation [ko00363], and metabolism of other amino acids such as glutathione metabolism [ko00480]. The central pathways identified as enriched for the downregulated transcripts were involved in carbohydrate metabolism including pentose and glucuronate interconversions [ko00400], starch and sucrose metabolism [ko00500], amino sugar and nucleotide sugar metabolism [ko00520] (Table 3).

**Table 2:** Enriched pathways of the up-regulated DEGs.

Pathway	Id	Sample number	Background number	P-Value	Corrected P-Value
Drug metabolism - cytochrome P450	ko00982	16	65	4.98E-10	1.29E-07
Metabolism of xenobiotics by cytochrome P450	ko00980	15	64	3.61E-09	4.66E-07
Glutathione metabolism	ko00480	20	163	1.01E-06	6.49E-05
Bisphenol degradation	ko00363	13	84	6.10E-06	0.000225
Polycyclic aromatic hydrocarbon degradation	ko00624	14	99	7.99E-06	0.000258
Aminobenzoate degradation	ko00627	14	102	1.14E-05	0.000326
Limonene and pinene degradation	ko00903	15	122	2.15E-05	0.000556
Tryptophan metabolism	ko00380	14	119	6.56E-05	0.001539
Stilbenoid, diarylheptanoid and gingerol biosynthesis	ko00945	12	104	0.000257	0.004741
Methane metabolism	ko00680	19	230	0.000443	0.007622
Glucosinolate biosynthesis	ko00966	6	36	0.001357	0.020595
Sphingolipid metabolism	ko00600	7	50	0.00159	0.022791
Galactose metabolism	ko00052	11	114	0.002032	0.027596

**Table 3:** Enriched pathways of the down-regulated DEGs.

Pathway	Id	Sample number	Background number	P-Value	CorrectedP-Value
Pentose and glucuronate interconversions	ko00040	26	104	1.34E-14	4.49E-12
Starch and sucrose metabolism	ko00500	37	369	1.07E-07	1.79E-05
Gap junction	ko04540	10	44	4.85E-06	0.000405
Pathogenic Escherichia coli infection	ko05130	11	66	3.78E-05	0.002524
Glucosinolate biosynthesis	ko00966	8	36	5.17E-05	0.002879
Amino sugar and nucleotide sugar metabolism	ko00520	27	355	0.000594	0.022046

### Validation of Illumina Expression Patterns by qRT-PCR Analysis

To confirm the reliability of the Solexa analysis, 22 candidate DEGs were selected and their expression was detected by qRT-PCR. The expression patterns from qRT-PCR showed general agreement with those from the Solexa sequencing (Table 4). The discrepancies with respect to ratio should be attributed to the essentially different algorithms and sensitivity between the two techniques [41]–[42]. In the analysis of gene expression profiling, the deep-sequencing method generated absolute rather than relative expression measurements.

**Table 4:** Validation of the RNA-Seq expression profiles of selected DEGs by qRT-PCR.

Transcript ID	Description	RNA-Seq(FPKM)			qRT-PCR
		CK	Pb1000	Log FC	Pb1000/CK
comp7581_c0_seq1	*HP4* no annotation	0	77.93	36.999341	2.62
comp7904_c0_seq1	*NAC29* NAC domain-containing protein 29	0	33.42	35.500309	222.86
comp13297_c0_seq1	*EFR088-2* ethylene-responsive transcription factor	0	26.67	34.839795	56.49
comp12162_c0_seq1	*LAA* long-chain acyl-CoA synthetase	0	15.31	34.74358	97.68
comp25182_c0_seq1	*LEAD-1* late embryogenesis abundant hydroxyproline-rich glycoprotei	0	10.05	33.74358	33.13
comp2239_c0_seq1	*GSTU11* glutathione-s-transferase	0.67	193.06	8.1793185	56.49
comp5087_c0_seq1	*NDR1* NDR1/HIN1-like 25salicylic acid	0.23	52.2	7.8221293	113.77
comp5862_c0_seq1	*GST1-1* glutathione S-transferase	0.89	44.35	5.6316074	10.85
comp1331_c0_seq1	*GST3-1*glutathione-s-transferase 3	4.86	114.66	4.5584999	10.13
comp2192_c0_seq1	*GGT1* gamma-glutamyl transpeptidase 1	4.82	107.13	4.4724431	8.28
comp9962_c0_seq1	*ASA* Ascorbate Peroxidase	2.89	38.79	3.7457784	5.24
comp932_c0_seq1	*GST1-2*glutathione S-transferase	7.61	100.68	3.7247168	7.62
comp2560_c0_seq1	*GSTL1* glutathione transferase lambda 1	6.33	82.32	3.7008077	776.05
comp942_c0_seq1	GST3-2 glutathione-s-transferase 3	6.36	75.85	3.5758534	17.15
comp674_c0_seq1	*GSHB1* glutathione synthetase	51.37	454.81	3.1451889	3.25
comp630_c0_seq1	*LAX2-1* auxin transporter-like protein 2	327.47	45.41	−2.85144	0.37
comp5751_c0_seq1	*SPDS* spermine synthase	54.48	4.7	−3.535811	0.02
comp8656_c0_seq1	*LAX2-2* auxin transporter-like protein 2	60.46	1.83	−5.049527	0.03
comp3052_c0_seq1	*LAX3* auxin transporter-like protein 3	199.38	4.81	−5.375419	0.13
comp4226_c0_seq1	*GSTF11* glutathione S-transferase F1	94.71	0	−36.5882	0.01
comp5860_c0_seq1	*SSF* sodium symporter family protein	79.66	0	−37.17514	0.05
comp1679_c0_seq1	*CCD8* carotenoid cleavage dioxygenase 8	180.13	0	−38.81567	0.01

**Figure 5:**
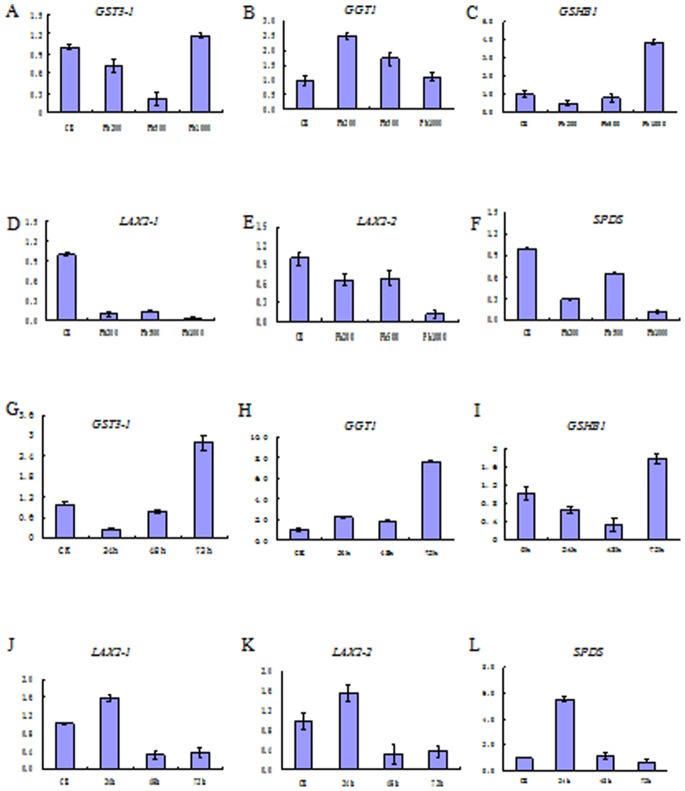
qRT-PCR analysis of six selected DEGs with different concentrations and temporal treatments of Pb(NO_3_)_2_. A-F represent different concentrations of Pb(NO_3_)_2_ after 72 h treatment and G-L represent different temporal duration of a fixed concentration of Pb(NO_3_)_2_ at 1000 mg L^−1^.

For further investigating and verifying the expression variations of the DEGs, transcriptional qRT-PCR analysis of six selected genes including three upregulated (*GST3-1*, *GGT1* and *GSHB1*) and three downregulated (*SPDS, LAX2-1* and *LAX2-2*), were performed for detailed analysis of different concentrations of Pb(NO_3_)_2_ (0, 200, 500 and 1000 mg L^−1^) after 72 h treatment and temporal duration (0, 24, 48 and 72 h) of a fixed concentration of Pb(NO_3_)_2_ at 1000 mg L^−1^. As shown in Figure 5, three downregulated DEGs, including *SPDS, LAX2-1* and *LAX2-2,* were downregulated under all the concentrations of Pb(NO_3_)_2_ treatments after 72 h, however, their expression exhibited variations among different time durations, which showed an upregulation for *SPDS* after 24 and 48 h, and *LAX2-1* after 24 h, while the remaining expressions were all downregulated. With an increase in Pb treatment duration, the levels of *SPDS*, *LAX2-1* and *LAX2-2* were gradually brought down, except for *LAX2-1* within 48 and 72 h where there was a slight increase. On the other hand, for glutathione metabolism upregulated genes, *GST3-1* and *GSHB1* showed downregulation to Pb(NO_3_)_2_ exposure for 200 and 500 mg L^−1^, but upregulation for 1000 mg L^−1^, whereas *GGT1* showed upregulation among all concentration treatments after 72 h. Meanwhile, the expression of these three genes showed significant changes at different duration treatments. *GST3-1*and *GSHB1* showed an upregulation at 24 and 72 h, respectively, while *GGT1* showed an increase at every exposure time point.

### Identification of DEGs Involved in Signal Sensing and Transduction Proteins

The main pathway by which heavy metal can penetrate into the plants is through root uptake from soils, and the root cell wall is directly in contact with metals in the soil solution [31], [43]. When encounter an extra-cellular stimuli, the cell walls could activate a variety of specific stress-responsive signaling proteins to protect the cell from susceptible sites into the protoplast, such as Mitogen-activated protein kinases (MAPKs) and calcium-binding related protein kinase. In eukaryotes, MAPKs were consisted of three sequentially activated protein kinases including MAPK kinase kinase (MAPKKK), MAPK kinase (MAPKK), and MAPK, all of which were involved in response to a variety of environmental, hormonal and developmental stimuli [31]. In this study, four DEGs were identified that were highly homologous to genes encoding MAPKs such as MAPKKK7, MAPK6, MAPK18 and MPAK20, and 14 DEGs were similar to calcium-binding protein genes including *CDPK9*, *CML45*, *CML22* and *EICBP5,* and most of these genes were upregulated under Pb stress (Table S4).

### Identification of DEGs Involved in Transcription Factors

Transcription factors (TFs) could regulate corresponding transcriptional processes when plants were treated with toxic heavy metals [30]. Numerous TFs such as WRKY, basic leucine zipper (bZIP), ethylene-responsive factor (ERF) and myeloblastosis protein (MYB) have been verified to play a significant role in controlling the expression of specific stress-related genes [44]–[45]. In this study, there were 58 DGEs including up- and down-regulated genes that were involved in different kinds of TFs such as the *WRKY* family (i.e., *WRKY4, 6, 28, 32, 33* and *75*), *ERF* family (i.e. *ERF1B*, *018*, *060*, *070*, *088* and *114*), *MYB family* (i.e., *MYB 1*, *28*, *29*, *46*, *51*, *59*, *95*, *116* and *122*), and dehydration-responsive element-binding protein-type transcription factors (*DREB*) (Table S4).

### Identification of DEGs Involved in Metal Transporters

Metal transporters could play a vital role in alleviating heavy metal toxicity by transporting metal ions out of the cell or sequestering it into the vacuole. It was reported that a wide range of transporter families including ATP binding cassette (ABC), Natural resistance-associated macrophage proteins (Nramps), ZRT, IRT-like proteins (ZIPs) and the Cation diffusion facilitators (CDFs), may contribute to heavy metal resistance [46]–[47]. In this study, 30 DGEs were identified as candidate genes involved as members of different metal transporter families, which were mainly related to ABC and ZIP families (Table S4).

### Identification of Candidate Genes Involved in Biosynthesis of Chelating Compounds and Glutathione Metabolism

Synthesis of Metal-chelating compounds is another mechanism used by plants to combat heavy metal stress, which can sequester and ultimately detoxify the excess metal ions [48]. Metallothioneins (MTs) are low-molecular-weight cysteine-rich metal binding peptides, which were usually classified into four groups (MT1–4). Currently, MT genes have been identified in a number of higher plants such as *Arabidopsis* and *Brassica juncea* [48]–[49]. In the present study, three DEGs were found homologs with genes encoding MT3. Phytochelatins (PCs) is another important class of heavy-metal-binding ligands, which can bind metal ions via thiolate coordination. PCs are not formed as a direct result of expression of a metal tolerance gene, but rather as a product of a biosynthetic pathway [50].Numerous physiological, biochemical and genetic studies have confirmed that glutathione (GSH) is the substrate for PC biosynthesis [51]. The conversion of GSH to PCs can be catalyzed by a special γ-glutamyl cysteine dipeptidyl transpeptidase (EC 2.3.2.15) called Phytochelatin sythase (PCS), and many genes involved in the GSH metabolism pathway [ko00480] have been found crucial in regulating GSH and PCs levels [52].

**Figure 6:**
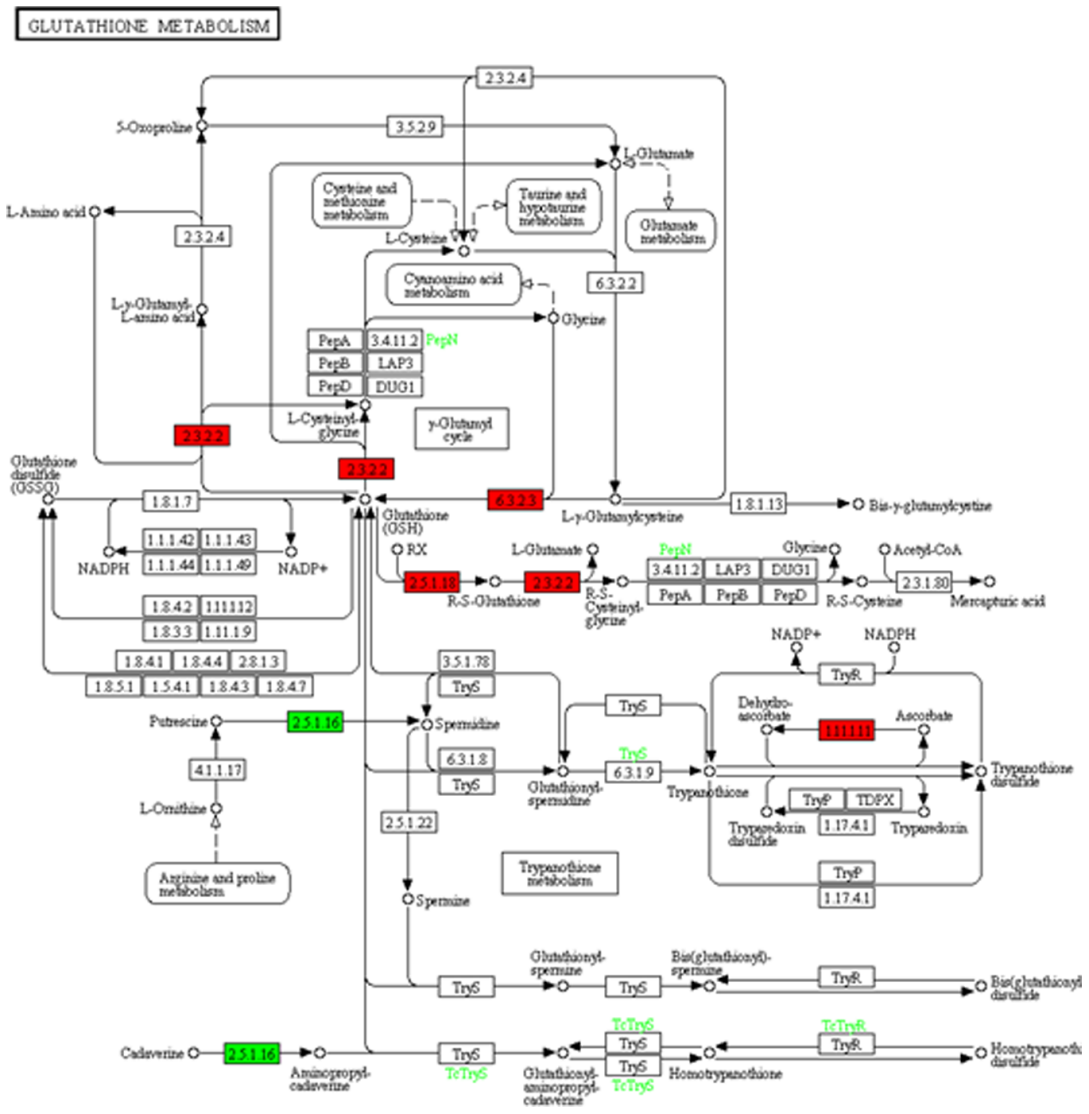
Expression pattern of genes involved in glutathione metabolism. The red and green color in each column indicated up- and down-regulation enzymes. The heatmaps generated were colored to the corresponding DEGs from the KEGG database (map 00480).

In the present study, only one DEG sequence was discovered encoding PCS. Based on the KEGG pathway assignment, 163 transcript sequences were found to encode 18 putative enzymes involved in glutathione metabolism from the present assembled *de novo* transcriptome background (Table 5, S1). More than one transcript sequences were annotated as the same enzyme, implying that such transcript sequences may represent different fragments of a single transcript, or different members of a gene family [53]. Comparative analysis of sequences from the two cDNA libraries during lead stress revealed that 25 transcript sequences were significantly differentially expressed. These sequences encoded five enzymes including four upregulated, L-ascorbate peroxidase (APX, EC 1.11.1.11), glutathione S-transferase (GST, EC 2.5.1.18), glutathione synthase (GSHB, EC 6.3.2.3) and gamma-glutamyltranspeptidase (GGT, EC 2.3.2.2), and one downregulated spermidine synthase (SPDS, EC 2.5.1.16) (Figure 6).

**Table 5:** The identified genes involved in glutathione metabolism of the *de novo* transcriptome.

KO No.	Gene description	Gene Name	EC Number
K00434	L-ascorbate peroxidase	*APX*	1.11.1.11
K00799	glutathione S-transferase	*GST*	2.5.1.18
K01920	glutathione synthase	*GSHB*	6.3.2.3
K00681	gamma-glutamyltranspeptidase	*GGT*	2.3.2.2
K00797	spermidine synthase	*SPDS*	2.5.1.16
K01469	5-oxoprolinase (ATP-hydrolysing)	*OPLAH*	3.5.2.9
K11205	glutamate–cysteine ligase regulatory subunit	*GCLM*	N/A
K01919	glutamate–cysteine ligase	*GSHA*	6.3.2.2
K01255	leucyl aminopeptidase	*PEPA*	3.4.11.1
K01256	aminopeptidase N	*PEPN*	3.4.11.2
K15428	Cys-Gly metallodipeptidase DUG1	*DUG1*	3.4.13.-
K00383	glutathione reductase (NADPH)	*GSR*	1.8.1.7
K00031	isocitrate dehydrogenase	*IDH1,*	1.1.1.42
K00033	6-phosphogluconate dehydrogenase	*PGD*	1.1.1.44
K00036	glucose-6-phosphate 1-dehydrogenase	*G6PD*	1.1.1.49
K00432	glutathione peroxidase	*GPX*	1.11.1.9
K10807	ribonucleoside-diphosphate reductase subunit M1	*RRM1*	1.17.4.1
K10808	ribonucleoside-diphosphate reductase subunit M2	*RRM2*	1.17.4.1

KO (KEGG Orthology), a classification of ortholog and paralog groups based on highly confident sequence similarity scores, and the reaction classification system for biochemical reaction classification, along with other classifications for compounds and drug.

## Discussion

Lead (Pb) is one of the most abundant, ubiquitous, toxic heavy metal elements posing a critical concern to human and environmental health [54]–[56]. Therefore, how to control Pb pollution and reduce Pb risks to human health has been an important health issue all over the world. Understanding the molecular mechanisms of plants responding to heavy metal stresses to control its uptake, translocation, and accumulation has been a focus work for plant genetic manipulation and biotechnology research [30], [57].

Radish (*Raphanus sativus* L.) is a major root vegetable crop worldwide, which can accumulate a relatively high concentration of toxic heavy metal elements, such as lead (Pb) and cadmium (Cd) in roots when grown in heavy metal polluted conditions [14]–[16]. However, few works have been reported about the Pb-responsive genes and their regulation networks in radishes under Pb stress.

Because the genome sequence of the radish is still unavailable, expressed sequence tag (EST) analysis has become one of the most valuable tools to discover and identify novel genes. However, to date, only 31, 4823 radish ESTs can be retrieved from the GenBank of NCBI, which has a limited coverage of the transcriptome, and only a few candidate genes involved in complex biosynthetic pathways can be identified. The arrival of Next-Generation Sequencing (NGS) technologies could overcome the limitations and allow investigators to simultaneously measure the changes and regulation at a genome-wide level under certain biological conditions for non-model plants for which the background genomic information is not available [39], [58]–[59]. In recent years,the newly developed NGS-based RNA-seq technique has been widely used for *de novo* transcriptome sequencing assembly, discovery of novel genes and investigation of gene expression in many non-model plants such as rice [60], peanuts [61], purple sweet potato [62], medicinal plant *Polygonum cuspidatum* [63] and floral plant *Dendrocalamus latiflorus* [64].

In this study, based on *de novo* transcriptome sequencing and assembly, a total of 68,940 assembled transcripts including 33,337 unigenes were obtained from two radish root cDNA libraries of untreated control (CK) and Pb-treated with Pb(NO_3_)_2_ at 1000 mg L^−1^ (Pb1000). Comparison of the assembled transcripts to gene catalogs of other species, 48,864 transcript sequences (70.88% of 68,940 transcripts) were annotated by BLAST analysis and functional bioinformatics analyses including the GO, COG and KEGG databases. From the annotation results, it could be found that the main species with BLAST hits to annotated transcripts were *A. thaliana*, *A. lyrata*, *B. napus*, *B. oleracea* and *B. rapa*, which indicated that the sequences of the radish transcripts obtained in the present study were assembled and annotated correctly [65]. However, an abundance of unannotated transcripts were generated in the present study, which may represent a specific gene pool for radish root studies. The gene catalog provided a comprehensive understanding of the gene transcription profiles of radishes, and laid a solid foundation for further investigation of Pb-stress mechanisms and identification of novel genes in this species.

Gene expression analysis is often employed in exploring changes among different plant tissues, different developmental stages, or plants under different treatments [20], [66]–[68]. In this study, a total of 4,614 transcripts were identified as differentially expressed genes (DEGs). By analysis of the statistically enriched GO terms and pathways related to the DEGs, it was revealed that the most significant and high frequently enriched terms and pathways for upregulated transcripts during Pb stress were predominately involved in the defense response in cell walls (GO:0052544 and GO:0052482) and glutathione (GSH) metabolism-related (i.e., GO:0006803, GO:0004364, and ko00480) processes, while for downregulated genes, they were mainly related to carbohydrate metabolism-related pathways (i.e., ko00400, ko00500 and ko00520) (Table 3, S2 and S3). This information could offer more direct biological insights to elucidate the response mechanism of Pb stress in radishes, which was in high accordance with previous studies of heavy metal stresses in plants [69]–[71].

The plant cell wall played an important role for heavy metal defense and detoxification, which could act as a barrier limiting metal uptake and penetration into the protoplast [43]. With electron microscopy observation, Inoue et al [15] reported that the presence of Pb in the cell wall of radish roots could form extremely large crystalline-like deposits, which can completely saturate the cell wall and need special accommodation space or thickening of the cell wall at the site. These findings supported the roles of cell wall-related terms ‘defense response by callose deposition in cell wall’ (GO: 0052544) and ‘defense response by cell wall thickening’ (GO: 0052482) identified in the present study following Pb stress in radishes. Additionally, plant cell walls have been consided as active metabolic sites, where a variety of specific stress-responsive signaling molecules such as mitogen-activated protein kinases (MAPKs) and calcium-binding related protein kinase could be generated in response to extra-cellular stimuli [31], [72]. Many studies from different plant species indicated that several MAPK pathways were activated in response to heavy metal stress. A previous screen in *Arabidopsis* for cadmium-responsive genes identification revealed that a related MAPK named MAPKKKMEK K1 could be induced by high concentrations of CdCl_2_ at transcriptional level [73]. Jonak et al (2004) reported that exposure of alfalfa (*Medicago sativa*) seedlings to excess copper or cadmium ions could activated four distinct MAPKs [74]. Currently, four DEGs were identified as MAPKs including MAPKKK7, MAPK6, MAPK18 and MPAK20 in response to Pb treatment in radish. Because the activation of MAPK cascade is plant-species and metal variety-dependent, the role of distinct MAPK modules in radish responding to Pb stress still to be verified for further study. Calmodulin system was reported to be involved in sensing heavy metal stress such as Cd, Ni and Pb, which could regulate ion transport, gene expression and stress tolerance [31]. The different gene expression pattern in our study showed that 14 DEGs were found homologous encoding calmodulin-like protein including both up and down regulated following Pb stress.

It was reported that GSH played a pivotal role in protecting plants from lead stress by quenching lead-induced reactive oxygen species (ROS). Liu et al. [34] by genome-wide microarray expression profiling analysis revealed that lead treatment could induce different GSH genes in *Arabidopsis thaliana*. Estrella-Gomez et al. indicated that lead accumulation in *Salvinia minima* could increase the accumulation of GSH, the enzymatic activity of glutathione synthase (GS), and cause expression changes of *SmGS* genes in both leaf and root tissues [75]. In addition Gupta et al. also reported the role of GSH in lead detoxification in *S. alfredii* [76]. In the present study, 18 GSH-related enzymes were found based on assembled radish transcriptome background and five of them were identified as DEGs including four upregulated and one downregulated under Pb stress (Table 5, Figure 6). The different gene expression showed a similar result to the previous reports. Moreover, many kinds of transcription factors (TFs), metal transporters, and metal-binding ligands have been found to be responsive and defensive to Pb stress in many other species [43]. As expected, these related gene families were also successfully identified in our study.

In summary, Next Generation Sequencing (NGS)-based RNA-seq technology was firstly employed in this study to characterize the roots *de novo* transcriptome of the radish plant under Pb stress, and a total of 68,940 assembled unique transcripts representing 33,337 unigenes were obtained. Based on the assembled *de novo* transcriptome, 4,614 differentially expressed genes (DEGs) that play significant roles in the response to Pb stress were identified. Gene Ontology (GO) and pathway enrichment analysis revealed that the upregulated DEGs under Pb stress were predominately involved in defense responses in the cell wall and glutathione metabolism-related processes, while downregulated DEGs were mainly related to carbohydrate metabolism pathways. Multiple candidate genes involved in defense and detoxification were successfully identified in response to Pb stress, and expression patterns of selected DEGs were further validated with qRT-PCR, which reflected significant alterations in major biological processes and metabolic pathways during Pb stress. The molecular basis of the response to Pb stress in radishes was first comprehensively characterized in this study, which resulted in useful information and laid a solid foundation for future investigating the molecular regulation mechanism of heavy metal Pb accumulation and tolerance in root vegetable crops.

## Materials and Methods

### Plant Materials

The radish *(Raphanus sativus* L.) advanced inbred line, ‘NAU-RG’, was used in this study. The surface-sterilized seeds were sown into soil in plastic pots and the seedlings were cultured in a growth chamber with 14 h light at 25°C and 10 h dark at 18°C for 20 days. Seedlings with similar sizes were transplanted into a 20-L plastic container with modified half-strength Hoagland’s nutrient solution (pH 5.4). One week later, the plants were treated with Pb(NO_3_)_2_ at 0 and 1000 mg L^−1^, respectively, and cultured under glasshouse conditions with natural light and day/night temperature of 28/18°C. For Solexa analysis and qRT-PCR verification, plants were harvested when they were treated with Pb(NO_3_)_2_ at 0 and 1000 mg L^−1^after 72 h. For qRT-PCR analysis of the different concentrations and temporal variation of Pb treatments, plants were collected after 72 h with different concentrations of Pb(NO_3_)_2_ at 0, 200 and 500 mg L^−1^, and for the concentration at 1000 mg L^−1^ were collected after 0, 24, 48, and 72 h, respectively. All samples were washed with deionized water and immediately frozen in liquid nitrogen and stored at −80°C for RNA extraction.

### RNA Isolation and Illumina Sequencing

Total RNA from different samples was isolated using the RNAprep pure Plant Kit according to the manufacturer’s protocol (Tiangen Biotech Co., Ltd., China). To avoid genomic DNA contamination, RNA samples were treated with RNase-free DNase I (Takara, Japan). Two radish root cDNA libraries were constructed using an mRNA-seq assay for paired-end transcriptome sequencing, which was performed by the Shanghai Majorbio Biopharm Technology Co., Ltd. (Shanghai, China). Poly(A) mRNA was enriched from total RNA by using Sera-mag Magnetic Oligo (dT) Beads (Thermo Fisher Scientific, USA) and then mRNA-enriched RNAs were chemically fragmented to short pieces using 1× fragmentation solution (Ambion, USA) for 2.5 min at 94°C. Double-stranded cDNA was generated using the SuperScript Double-Stranded cDNA Synthesis Kit (Invitrogen, USA). After that, the Illumina Paired End Sample Prep kit was used for RNA-seq library construction and then was sequenced using Illumina HiSeq™ 2000. The sequencing data were deposited in NCBI Sequence Read Archive (SRA, http://www.ncbi.nlm.nih.gov/Traces/sra) with accession numbers SRX256970 (CK) and SRX263753 (Pb1000).

### Raw Sequence Processing and *de novo* Assembly

Raw reads generated by Illumina Hiseq^TM^2000 were initially processed to get clean reads by removing the adapter sequences and low quality bases at the 3' end. Then, transcriptome *de novo* assembly was carried out using the short-read assembling program Trinity [35]. First, clean reads with a certain length of overlap were combined to form longer contiguous sequences (contigs), and then these reads were mapped back onto the contigs. The distance and relation among these contigs could be calculated based on paired-end reads, which enabled the detection of contigs from the same transcript and also calculation of the distances among these contigs. Finally, contigs were further assembled using Trinity, and the contigs that could not be extended on either end were defined as unique transcripts. Additionally, the unique assembled transcripts were further subjected to the process of sequence-splicing redundancy removal with sequence clustering software to acquire non-redundant transcripts called unigenes.

### Functional Annotation of the Assembled Transcripts

All the assembled transcripts were searched against the NCBI non-redundant (nr) Swiss-Prot, COG and KEGG databases using BLASTX to identify the proteins that had the highest sequence similarity with the given transcripts to retrieve their function annotations and a typical cut-off E-value of <10^−5^ was set. For the nr annotations, the BLAST2GO program was used to get GO annotations of unique assembled transcripts for describing biological processes, molecular functions and cellular components [77] After getting GO annotations for each transcript, WEGO software [78] was used to conduct GO functional classification for understanding the distribution of gene functions at the macroscopic level.

### Identification and Functional Enrichment Analysis of the Differentially Expressed Genes (DEGs)

To identify DEGs between the two different treatments, the expression level for each transcript was calculated using the fragments per kilobase of exon per million mapped reads (FRKM) method. R statistical package software edgeR (Empirical analysis of Digital Gene Expression in R) was employed to quantify differential gene expression [79]. In addition, functional-enrichment analysis including GO and KEGG were performed to identify which DEGs were significantly enriched in GO terms and metabolic pathways at Bonferroni-corrected P-value ≤0.05 compared with the whole-transcriptome background using the formula described in the previous studies [24], [42].

### Validation of DEG Expression with Quantitative Real-time PCR (qRT-PCR)

Quantitative real-time PCR was performed on a MyiQ Real-Time PCR Detection System (Bio-Rad) platform using the SYBR Green Master ROX (Roche, Japan) following the manufacturer’s instructions. Primers were designed using Beacon Designer 7.0 software, and Actin2/7 (*ACT*) (Table S5) was selected as the internal control gene according to a previous report [80]. The amplification was achieved by the following PCR program of first denaturation 95°C for 3 min, then 40 cycles of denaturation at 95°C for 5 s, annealing and extension at 58°C [62], [80]. The relative expression levels of the selected transcripts normalized to *ACT* were calculated using the 2^−ΔΔCt^ method. All reactions were performed with three replicates, and the data were analyzed using Bio-Rad CFX Manager software.

## Supporting Information

Table S1 **KEGG pathways of the assembled transcripts.**
(XLS)

Table S2 **The enriched GO terms of up-regulated DEGs.**
(XLS)

Table S3 **The enriched GO terms of down-regulated DEGs.**
(XLS)

Table S4 **Candidate genes involved in defense and detoxification mechanisms of radish roots in repose to heavy metal Pb.**
(DOC)

Table S5 **Primers used for qRT-PCR.**
(XLS)
