# Supplementary material for: Transcriptome Profiling of Radish (Raphanus sativus L.) Root and Identification of Genes Involved in Response to Lead (Pb) Stress with Next Generation Sequencing
Source: PLoS One. 2013 Jun 20;8(6):e66539. doi: 10.1371/journal.pone.0066539 (PMC3688795; doi:10.1371/journal.pone.0066539)
Supplement: Table S4 — Candidate genes involved in defense and detoxification mechanisms of radish roots in repose to heavy metal Pb. (DOC) [file pone.0066539.s004.doc]

**Table S4 .Candidate genes involved in defense and detoxification mechanisms of radish roots in repose to heavy metal Pb**

| **Transcript ID LogFC** | | **PValue** | **FDR** | **Description** |
| --- | --- | --- | --- | --- |
| **Signaling protein kinases** | | | | |
| comp32129_c0_seq1 | 34.35511 | 7.45E-07 | 1.55E-05 | Mitogen-activated protein kinasekinase kinase 7 MAPKKK7 |
| comp30967_c0_seq1 | -4.92092 | 8.44E-05 | 0.000793 | double MYC-tagged mitogen activated protein kinase kinase 6 |
| comp29181_c0_seq5 | -35.033 | 1.47E-08 | 5.80E-07 | Mitogen-activated protein kinase 20 |
| comp28629_c0_seq2 | -35.1753 | 6.40E-09 | 2.90E-07 | Mitogen-activated protein kinase 18 |
| comp20510_c0_seq2 | -33.6 | 2.82E-05 | 0.000314 | Calmodulin binding protein-like protein |
| comp20510_c0_seq3 | -33.8917 | 7.72E-06 | 0.000108 | Calmodulin binding protein-like protein |
| comp22272_c0_seq2 | -33.4587 | 5.79E-05 | 0.000571 | putative calcium-binding protein CML22 |
| comp22703_c0_seq1 | -36.3656 | 3.93E-12 | 6.26E-10 | putative calcium-binding protein CML36 |
| comp25718_c0_seq1 | 4.214724 | 7.93E-08 | 2.36E-06 | Calmodulin-binding-like protein |
| comp25718_c0_seq4 | 5.101172 | 1.01E-07 | 2.89E-06 | Calmodulin-binding protein |
| comp25718_c0_seq8 | 7.149466 | 4.57E-12 | 7.07E-10 | Calmodulin-binding protein |
| comp26204_c0_seq1 | 5.651303 | 1.52E-07 | 4.05E-06 | putative calcium-binding protein CML45 |
| comp27958_c0_seq4 | 3.361623 | 2.10E-05 | 0.000251 | Calmodulin-domain protein kinase CDPK isoform 9 |
| comp27958_c0_seq7 | 33.63215 | 2.82E-05 | 0.000314 | Calmodulin-domain protein kinase CDPK isoform 9 |
| comp28722_c0_seq1 | 3.457689 | 5.56E-05 | 0.000564 | Calmodulin binding protein IQD22 |
| comp31729_c1_seq4 | -33.8686 | 7.72E-06 | 0.000108 | Calmodulin-binding protein |
| comp31732_c2_seq1 | -33.7218 | 1.80E-05 | 0.000217 | Calmodulin-binding transcription activator 5 |
| comp48470_c0_seq1 | 34.35159 | 7.45E-07 | 1.55E-05 | Calmodulin-binding protein |
| **Modulation of transcription factors** | | | | |
| comp31859_c1_seq3 | -33.6608 | 2.25E-05 | 0.00026 | bZIP transcription factor family protein |
| comp28781_c0_seq1 | -33.3323 | 9.64E-05 | 0.000873 | Similar to bZIP transcription factors |
| comp12604_c0_seq1 | 33.50359 | 4.53E-05 | 0.000467 | WRKY DNA-binding protein 31 |
| comp12604_c1_seq1 | 33.85974 | 9.48E-06 | 0.000129 | WRKY DNA-binding protein 31 |
| comp20309_c0_seq2 | 6.678571 | 2.51E-08 | 9.07E-07 | WRKY75-1 transcription factor |
| comp25906_c0_seq2 | -3.825 | 3.56E-05 | 0.000382 | WRKY transcription factor 13 |
| comp26560_c0_seq3 | -3.34794 | 5.85E-05 | 0.000576 | WRKY family transcription factor |
| comp26773_c0_seq1 | 5.730495 | 5.79E-07 | 1.27E-05 | WRKY28-1 transcription factor |
| comp28993_c0_seq11 | -33.9955 | 4.28E-06 | 6.67E-05 | WRKY4-1 transcription factor |
| comp28993_c0_seq24 | 33.806 | 1.17E-05 | 0.000152 | WRKY4-1 transcription factor |
| comp29650_c1_seq11 | 37.04843 | 4.73E-14 | 1.55E-11 | WRKY33-1 transcription factor |
| comp29650_c1_seq14 | 12.55439 | 1.18E-12 | 2.24E-10 | WRKY33-1 transcription factor |
| comp29650_c1_seq20 | 33.69313 | 2.25E-05 | 0.00026 | WRKY33-1 transcription factor |
| comp29650_c1_seq21 | 4.511687 | 2.53E-06 | 4.29E-05 | WRKY33-1 transcription factor |
| comp29650_c1_seq4 | 5.423002 | 1.23E-06 | 2.34E-05 | WRKY33-1 transcription factor |
| comp29650_c1_seq6 | 5.667068 | 2.95E-09 | 1.57E-07 | WRKY33-1 transcription factor |
| comp30458_c0_seq3 | -34.5489 | 2.23E-07 | 5.58E-06 | WRKY DNA-binding protein 32 |
| comp30458_c0_seq4 | 5.845161 | 1.14E-05 | 0.000152 | WRKY32-1 transcription factor |
| comp33895_c0_seq1 | 5.235651 | 2.80E-10 | 2.13E-08 | WRKY6-1 transcription factor |
| comp10969_c1_seq1 | 4.267253 | 4.23E-05 | 0.000447 | ethylene-responsive transcription factor 1B |
| comp17364_c0_seq1 | 4.667791 | 3.09E-05 | 0.000341 | ethylene-responsive transcription factor ERF114 |
| comp25838_c0_seq1 | -3.80446 | 1.47E-05 | 0.000184 | ethylene-responsive transcription factor ERF018 |
| comp27178_c0_seq2 | -33.7827 | 1.17E-05 | 0.000152 | ethylene-responsive transcription factor ERF070 |
| comp27178_c0_seq5 | -34.359 | 6.35E-07 | 1.37E-05 | ethylene-responsive transcription factor ERF070 |
| comp28742_c0_seq2 | 33.59201 | 3.57E-05 | 0.000382 | ethylene-responsive transcription factor ERF060 |
| comp37604_c0_seq1 | -4.27277 | 1.19E-05 | 0.000155 | ethylene-responsive transcription factor ERF018 |
| comp40344_c0_seq1 | 34.81393 | 5.90E-08 | 1.86E-06 | ethylene-responsive transcription factor ERF088 |
| comp10200_c0_seq1 | -34.8005 | 5.90E-08 | 1.86E-06 | MYB domain protein 28-2 |
| comp11171_c0_seq1 | 35.3833 | 1.90E-09 | 1.07E-07 | MYB domain protein 116 |
| comp13941_c0_seq1 | -3.69928 | 1.15E-05 | 0.000152 | MYB domain protein 58 |
| comp13941_c0_seq2 | -34.264 | 1.03E-06 | 2.03E-05 | MYB-related transcription factor |
| comp16974_c0_seq1 | 34.74238 | 8.64E-08 | 2.52E-06 | MYB domain protein 122 |
| comp19328_c0_seq1 | -4.06161 | 1.21E-05 | 0.000158 | MYB domain protein 95 |
| comp19625_c0_seq1 | 3.641319 | 5.98E-05 | 0.000587 | MYB proto-oncogene protein |
| comp20458_c0_seq1 | 33.71772 | 1.80E-05 | 0.000217 | MYB domain protein 108 |
| comp22094_c0_seq1 | 3.827091 | 2.07E-06 | 3.60E-05 | MYB domain protein 51-2 |
| comp22094_c0_seq2 | 7.446764 | 2.01E-12 | 3.60E-10 | MYB domain protein 51-1 |
| comp22841_c0_seq1 | -36.3403 | 4.76E-12 | 7.27E-10 | MYB domain protein 29-1 |
| comp23113_c0_seq3 | -3.69853 | 7.85E-05 | 0.000741 | MYB family transcription factor |
| comp23749_c0_seq4 | -33.3136 | 9.64E-05 | 0.000873 | MYB-related protein 1 |
| comp24841_c0_seq5 | -33.5535 | 3.57E-05 | 0.000382 | MYB family transcription factor |
| comp24841_c0_seq7 | -35.4309 | 1.34E-09 | 8.21E-08 | MYB family transcription factor |
| comp24970_c0_seq1 | -3.0438 | 9.26E-05 | 0.000863 | MYB domain protein 28-3 |
| comp24970_c0_seq2 | -35.7389 | 2.09E-10 | 1.67E-08 | MYB domain protein 28-3 |
| comp25516_c1_seq1 | -33.6888 | 1.80E-05 | 0.000217 | MYB family transcription factor |
| comp26013_c0_seq1 | -33.8883 | 7.72E-06 | 0.000108 | transcription factor MYB59 |
| comp27196_c0_seq2 | -34.3454 | 7.45E-07 | 1.55E-05 | MYB family transcription factor |
| comp28894_c0_seq10 | 34.05272 | 3.54E-06 | 5.71E-05 | MYB family transcription factor |
| comp28894_c0_seq13 | 11.09615 | 1.07E-08 | 4.48E-07 | MYB family transcription factor |
| comp28894_c0_seq14 | -33.8044 | 1.17E-05 | 0.000152 | MYB family transcription factor |
| comp29677_c0_seq1 | -4.09542 | 9.85E-06 | 0.000133 | AtMYB46 |
| comp29737_c0_seq1 | -33.8161 | 9.48E-06 | 0.000129 | MYB transcription factor |
| comp29815_c2_seq3 | -34.0117 | 4.28E-06 | 6.67E-05 | MYB-like HTH transcriptional regulator family protein |
| comp30492_c0_seq2 | -37.2933 | 9.02E-15 | 3.93E-12 | MYB transcription factor CDC5 |
| comp31511_c0_seq2 | -5.4361 | 2.21E-09 | 1.24E-07 | Dehydration-responsive family protein |
| comp31919_c1_seq23 | 34.25412 | 1.22E-06 | 2.33E-05 | Dehydration-responsive family protein |
| comp31919_c1_seq32 | 33.93784 | 6.32E-06 | 9.18E-05 | Dehydration-responsive family protein |
| comp31919_c1_seq33 | 33.95651 | 5.19E-06 | 7.80E-05 | Dehydration-responsive family protein |
| **Activation of metal transporters** | | | | |
| comp24735_c0_seq1 | -4.51956 | 1.98E-05 | 0.000236 | ABC transporter family protein |
| comp27368_c0_seq1 | 3.481386 | 2.38E-06 | 4.12E-05 | ABC transporter A family member 1 |
| comp28612_c0_seq1 | 3.011655 | 2.50E-05 | 0.000287 | ABC transporter A family member 2 |
| comp28654_c0_seq2 | -33.3518 | 9.64E-05 | 0.000873 | ABC transporter family protein |
| comp29837_c0_seq1 | 4.1056 | 6.82E-08 | 2.08E-06 | ABC1 family protein |
| comp30136_c0_seq31 | 5.433519 | 5.25E-07 | 1.17E-05 | ABC transporter G family member 37 |
| comp30451_c0_seq1 | 4.17218 | 2.61E-07 | 6.40E-06 | ABC transporter B family member 11; |
| comp30451_c1_seq1 | 4.081567 | 1.73E-07 | 4.51E-06 | ABC transporter B family member 21 |
| comp30451_c3_seq1 | 3.39003 | 3.79E-06 | 6.08E-05 | ABC transporter B family member 21 |
| comp31653_c1_seq13 | 34.21483 | 1.45E-06 | 2.66E-05 | ABC transporter G family member 31 |
| comp31657_c0_seq1 | -34.3193 | 7.45E-07 | 1.55E-05 | ABC transporter G-24 |
| comp31657_c0_seq2 | 4.523909 | 2.17E-08 | 8.04E-07 | ABC transporter G-24 |
| comp31946_c0_seq7 | -33.5074 | 4.53E-05 | 0.000467 | ABC transporter D family member 1 |
| comp32093_c0_seq1 | 37.97826 | 9.17E-17 | 8.25E-14 | ABC transporter C family member 10 |
| comp32112_c0_seq2 | 35.91676 | 7.15E-11 | 6.90E-09 | MRP-like ABC transporter |
| comp32112_c0_seq4 | 5.770723 | 1.45E-05 | 0.000181 | MRP-like ABC transporter |
| comp32175_c0_seq1 | -3.9668 | 1.11E-07 | 3.12E-06 | ABC transporter-like protein |
| comp32175_c0_seq3 | -3.23306 | 1.95E-05 | 0.000234 | ABC transporter B family member 1 |
| comp33322_c0_seq1 | -4.2268 | 2.34E-08 | 8.51E-07 | ABC transporter family protein |
| comp33573_c0_seq1 | 9.202713 | 1.50E-17 | 2.30E-14 | ABC transporter G family member 40 |
| comp21392_c1_seq1 | 5.20836 | 6.91E-05 | 0.000672 | homeobox-leucine zipper protein 9 |
| comp24261_c0_seq6 | -4.04323 | 8.63E-08 | 2.52E-06 | ZIP family metal transporter |
| comp24261_c0_seq7 | -2.98229 | 3.34E-05 | 0.000366 | ZIP-like zinc transporter |
| comp25068_c0_seq1 | -34.0089 | 4.28E-06 | 6.67E-05 | zinc transporter 3 |
| comp25068_c0_seq2 | -3.65671 | 2.74E-06 | 4.63E-05 | zinc transporter 3 |
| comp27425_c0_seq1 | -36.0061 | 3.86E-11 | 4.16E-09 | putative zinc transporter 10 |
| comp28781_c0_seq1 | -33.3323 | 9.64E-05 | 0.000873 | Similar to bZIP transcription factors |
| comp30001_c0_seq2 | -35.4442 | 1.34E-09 | 8.21E-08 | homeobox-leucine zipper protein ATHB-15 |
| comp30001_c0_seq4 | -33.5539 | 3.57E-05 | 0.000382 | homeobox-leucine zipper protein ATHB-15 |
| comp31848_c0_seq2 | -4.3347 | 1.80E-07 | 4.67E-06 | homeobox-leucine zipper protein ATHB-8 |
| **Biosynthesis of chelating compounds** | | | | |
| comp29319_c0_seq3 | 4.916962 | 1.71E-08 | 6.62E-07 | Phytochelatin synthase 1 |
| comp31628_c1_seq10 | -3.61844 | 4.19E-06 | 6.67E-05 | Metallothionein type 3 |
| comp31628_c1_seq7 | -37.9793 | 9.02E-17 | 8.25E-14 | Metallothionein type 3 |
| comp31628_c1_seq9 | -38.1598 | 2.64E-17 | 3.09E-14 | Metallothionein type 3 |
